# Effects of Traditional vs. iPad-Enhanced Aerobic Exercise on Wayfinding Efficacy and Cognition: A Pilot Randomized Controlled Trial

**DOI:** 10.3390/ijerph16183495

**Published:** 2019-09-19

**Authors:** Daniel Palac, Tiffany Bullard, Jason D. Cohen, Lydia T. Nguyen, Raksha A. Mudar, Sean P. Mullen

**Affiliations:** 1 Department of Kinesiology & Community Health, University of Illinois at Urbana-Champaign, Urbana, IL 61801, USA; palac1@illinois.edu (D.P.); bullard3@illinois.edu (T.B.); cohenj225@gmail.com (J.D.C.); 2 Neuroscience Program, University of Illinois at Urbana-Champaign, Urbana, IL 61801, USA; ltnguyn2@illinois.edu (L.T.N.); raksha@illinois.edu (R.A.M.); 3 Department of Speech and Hearing Science, University of Illinois at Urbana-Champaign; Champaign, IL 61820, USA; 4 Beckman Institute for Advanced Science and Technology, University of Illinois at Urbana-Champaign; Urbana, IL 61801, USA; 5 Illinois Informatics Institute, University of Illinois at Urbana-Champaign; Urbana, IL 61801, USA

**Keywords:** mHealth, life-space use, self-efficacy, cognitive function, physical activity

## Abstract

The purpose of this pilot study was to test the feasibility and efficacy of an iPad-enhanced aerobic exercise intervention designed to enhance wayfinding efficacy and performance and relevant cognitive functioning among middle-aged adults at risk for cognitive impairment. Twenty-seven low active adults (21 females) aged 45 to 62 years (51.22 ± 5.20) participated in a ten-week randomized controlled trial. Participants were randomized to an iPad-enhanced aerobic exercise group (experimental group) or an aerobic exercise-only group (control group) following baseline assessment. Both groups exercised at 50% to 75% of age-predicted heart rate maximum for 30 to 50 min/d, 2 d/wk for 10 weeks. During aerobic exercise, the experimental group engaged in virtual tours delivered via iPad. Baseline and post-intervention assessments of wayfinding self-efficacy, wayfinding task performance, cognitive functioning, electroencephalogram (EEG), and psychosocial questionnaires were administered. The results suggest that ten weeks of iPad-enhanced, moderately intense aerobic exercise had specific effects on wayfinding self-efficacy; however, no statistical differences were found between groups on the behavioral wayfinding task or spatial memory performance at follow-up. Performance scores on an inhibitory attentional-control cognitive assessment revealed significant differences between groups, favoring the experimental group (*p* < 0.05). Virtual reality-enhanced aerobic exercise may prove to be an effective method for improving cognitive function and increasing confidence to navigate real-world scenarios among individuals at risk of cognitive impairment.

## 1. Introduction

Wayfinding is “an interactive, problem-solving process by which people use environmental information to locate themselves and navigate from place to place” [1]. One’s wayfinding ability requires a wide range of cognitive abilities, including spatial knowledge, decision making, planning, and monitoring processes [2]. Numerous technologies, such as voice-guided Global Positioning Systems and electronic interactive display screens, such as those often found in shopping malls, can help orient one’s location and aid in determining the best route for reaching a destination [3]. Individuals use various strategies to navigate environments. These strategies often include the use of maps, landmarks, and signage. The wayfinding process consists of three stages: (1) formulating a plan or cognitive map relying on spatial information from the environment (e.g., landmarks) or from other sources (e.g., maps), (2) decision-making that guides wayfinding behavior (e.g., shortest path, fewest turns, most scenic), and (3) transforming decisions into actions [4]. Formulating a plan also requires route-planning elements such as ‘taking the elevator’ and knowing the location of the destination. From this plan, cognitive representations are used to execute actions.

Unsuccessful wayfinding is often the result of impaired spatial ability, which can lead to frustration and confusion [5]. Among young and older adults, lower performance on measures of visuo-spatial memory (immediate and delayed free recall and recognition tasks of the Visual Reproduction Test from the Wechsler Memory Scale) and visuo-spatial executive functioning (the Mental Rotation Test, Digit Symbol-Coding Test, and Trail Making Test Part B) has been associated with lower virtual reality-based wayfinding performance [6]. Significant age effects have been observed on wayfinding performance mediated by measures of executive function, such as performance on a dual-task paradigm [7]. Adding to this evidence, Poranen-Clark et al. [8] found evidence that performance on the Trail Making Test Parts A and B was a determinant of older adults’ ability to navigate environments over a two-year period. Age-related differences have also been observed when learning novel routes or exploring unfamiliar environments. Using virtual environments, Wiener and colleagues (2012) revealed age-related deficits in route retracing and overall travel direction when comparing performance of older (61–80 years) versus younger (25–30 years) adults. These deficits may be due to impaired allocentric navigation strategies, which are hippocampus dependent [9].

Mild cognitive impairment (MCI) refers to a specific cognitive status in which individuals experience cognitive decline above and beyond what is expected for their age and education but without significant functional impairment. MCI affects approximately 18.9% of adults who are at least 50 years old [10] and is considered to be a risk factor for Alzheimer’s disease (AD) and other dementia. Moreover, MCI is known to impact spatial knowledge and executive function, both of which are critical for wayfinding. In particular, amnestic MCI patients whose primary deficits are in memory functions tend to perform worse at egocentric navigation than healthy older adults [11] and have less volume of the right hippocampal subregions, which has been associated with poorer navigation skills, according to performance on visuoconstructive tasks (e.g., clock drawing [12]). Additionally, those with amnestic MCI have difficulty switching between frames of reference when navigating environments (i.e., egocentric to allocentric spatial representations). On an Ego-Allo-Switching spatial memory task, patients with amnestic MCI report less accurate verbal judgments about relative distances between memorized stimuli when compared to normal controls [13]. With more severe cognitive impairment with disease progression, there is an increased likelihood for changes and challenges with mobility and navigation in this population [14,15,16].

Previously, Mullen et al. [17] found that older adults’ (65+ years) confidence in wayfinding, among other outcomes, was positively associated with self-reported walking behavior. Thus, we considered creating a physical activity program to increase mobility and improve wayfinding behavior. To assess self-reported mobility in adult populations, researchers often rely on the Life-Space Assessment (LSA). This assessment measures mobility indirectly, in terms of where individuals travel and how often any assistance is required [18]. Higher composite scores on the LSA are indicative of greater appraisal of one’s mobility. Poor functional and cognitive performance has been associated with lower composite LSA scores [19,20,21]. Furthermore, life-space mobility has been associated with fall risk. As one’s life space mobility decreases, the risk for falls and subsequent injury increases [22]. Among 848 relatively healthy older adults, Portegijs and colleagues found that impaired physical performance along with a limited sense of autonomy for outdoor activities had independent associations with increased restrictions in life space mobility [20].

Evidence suggests that regular engagement in physical activity is associated with beneficial effects on brain function among older adults who are at risk for AD [23,24]. A recent meta-analytic review revealed aerobic exercise interventions as having a moderate-to-strong pooled effect size on cognition in AD studies and a small effect size in MCI studies [25]. Other specialized functional fitness training (e.g., balance and gait speed) among healthy older adults and those with MCI are relatively robust in terms of improving cognitive function [26], but given the systemic effects of aerobic training on executive functioning [27,28,29,30], such training is likely to be of the greatest utility to those at-risk of chronic cognitive decline. Considering the cognitive benefits of physical activity among middle-aged adults, targeting those who are at-risk for cognitive impairment might have the most beneficial outcomes as they relate to visuospatial cognition and wayfinding.

In order to deliver a more meaningful exercise experience and allow for greater cognitive gains, researchers have utilized exergames—reality-enhanced exercise that combines physical activity with computer-simulated environments [31,32]. Such interactive simulation experiences have become popular in promoting healthy behaviors [33]. An exergame’s controls are enacted via users’ body movements, and in some cases, have been shown to produce comparable energy expenditure levels with that of traditional exercise for middle-age and older adults [34]. Among adults with dementia, exergames are often perceived as enjoyable and accessible without assistance as they are generally able to remember game controls [35]. It is now possible to play these types of active videogames via traditional gaming consoles (e.g., Wii Fit) and smart devices (e.g., iPad tablet). Similarly, virtual reality (VR) platforms used in wayfinding training have been identified as helpful tools for stroke victims who have spatial anxiety, limited mobility, and impaired navigational skills [36].

Some VR platforms simulate natural environments to guide a user’s exercise gaming experience, which may be important because those who exercise outdoors tend to exhibit decreases in tension, confusion, anger, and depression compared to those who exercise indoors [37]. Anderson-Hanley et al. (2012) tested the efficacy of a cybercycling intervention involving outdoor-themed, VR-enhanced interactive exercise that yielded greater cognitive improvements among older adults with MCI compared to an intensity-matched cycling condition without VR [32]. In a follow-up study, Anderson-Hanley and colleagues tested a similar VR neuro-exergame system (with added cognitive challenges) with older adults, which revealed improvement in executive function following a single session [38]. In a secondary data analysis, Wall et al. found changes in their cortisol and insulin-like growth factor 1 that corresponded with improved executive function as measured by the Stroop task [39]. Increased engagement in exergaming was also associated with higher levels of brain-derived neurotrophic factor and increases in gray matter volume in the prefrontal cortex and anterior cingulate cortex. Finally, Mrakic-Sposta and colleagues (2018) found tendencies toward improvements on the Mini-Mental State Exam, a visual-constructive test, and a visuo-spatial test of attention among 10 participants with MCI after receiving six weeks of physical and cognitive training when compared to time-matched controls [40]. Together, these data suggest that older adults with and without MCI are responsive to this form of interactive exercise. Promising evidence gathered from Gadler et al. (2009) revealed improved performance in measures of attention and orientation between two individuals with MCI following four weeks of VR training focused on multitasking (e.g., navigating a supermarket for groceries) [41]. However, the benefits of exergaming on cognitive processes associated with navigation are still not well established.

The purpose of this study was to test a novel, 10-week iPad-enhanced aerobic exercise program on levels of physical activity, wayfinding efficacy, and cognitive functioning among adults screened for cognitive impairment. We hypothesized that the intervention group would have greater levels of physical activity, increased wayfinding self-efficacy and performance. Secondary aims included replicating previous work that utilized a VR-delivered naturalistic environment for physical activity and its effect on performance during a behavioral wayfinding task as well as measures of executive functioning and visuospatial memory.

## 2. Materials and Methods

### 2.1. Participants & Procedure

Middle-aged adults between 45 and 64 years of age with scores below 23 on the modified Telephone-Interview Cognitive Survey (TICS-m) were recruited between August 2014 and March 2016 via digital media outlets targeting residents living within a 20-mile radius of a large college campus in central Illinois. The TICS-m assesses global cognitive function and has been shown to have high reliability and validity as a cognitive status screening tool in differentiating normal cognition from MCI and dementia [42]. According to the original study, Welsh reported an MCI threshold score of 27 when using the 13-item TICS-m, which has a scoring range of 0–39, with higher scores indicating better cognitive performance [43]. Since then, researchers have suggested the importance of controlling for education when determining a cutoff score to distinguish between normal and cognitively impaired adults. As a result, Castanho recommends <23 be used in classifying participants with and without possible cognitive impairment [44]. A flyer and email advertisement directed participants to an online recruitment form. Research staff screened all participants via telephone. Exclusion criteria included diagnosed neurological disorders (e.g., dementia; Parkinson’s disease), functional disabilities that would inhibit participation in aerobic exercise, being physically active most days per week (>30 min/day) for the last three months, and a TICS-m score >23. Additionally, each participant must have received documented physician’s approval prior to participation in any baseline assessments. A university ethics board approved the study and all participants provided written informed consent.

**Figure 1:**
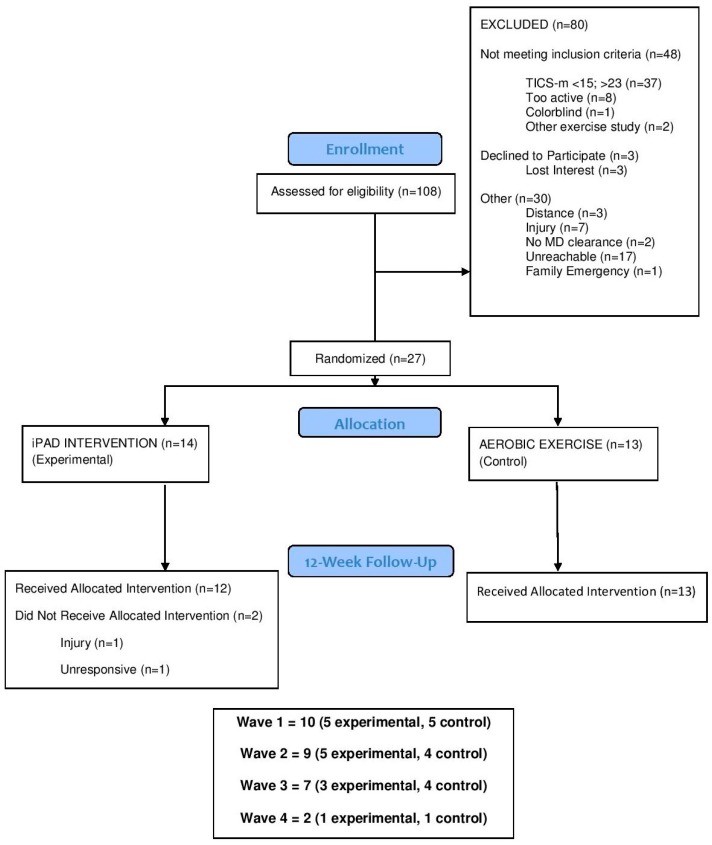
Flow chart of participant recruitment, randomization, and study completion.

One-hundred and eight individuals expressed interest in study participation. A total of 27 individuals met inclusionary criteria and were randomized into either the experimental or control group (see CONSORT in Figure 1). Out of the 27 participants randomized, 26 completed follow-up testing. There were no demographic differences (i.e., age, sex, baseline physical activity levels Table 1). Participants were mostly female (77.88%), Caucasian (66.7%), with a college degree (81.5%), and had a body mass index indicative of obesity (63%; *M* = 32.11, *SD* = 6.52; range = 19.50 to 48.10). Additionally, the majority were living with untreated pre-hypertension (63%; *M* systolic blood pressure [BP]/diastolic BP = 130.15/83.26 mmHg). Of the individuals living with pre-hypertension, 12% (*n* = 2) reported taking BP medication and none of the participants were on cholinesterase inhibitors. It is also worth noting that our sample appears to have wayfinding challenges, as indicated by their LSA score (*M* = 84.70, *SD* = 23.66; 44% spent less than 1 occasion per week out of town), low wayfinding self-efficacy (*M* = 63.49% [100% max score], *SD* = 31.86), and mobility (timed Up and Go performance: *M* = 5.26 *s*, *SD* = 0.98) at baseline, reflecting the multifaceted nature of the problem.

Participants completed psychosocial questionnaires that included items pertaining to wayfinding self-efficacy, a neuropsychological test battery, electroencephalogram (EEG) recordings during a strategic processing task and a real-world wayfinding task at baseline and follow-up. Trained research staff, blinded to group assignment, administered all testing assessments. At each participant’s initial visit, an accelerometer (Fitbit device) was provided to track physical activity for seven days. Participants were instructed to schedule their second visit at least one week following their first in order to allow for one full week of tracking. If participants completed their second appointment before one week had passed, they returned their accelerometer at a later date in order to allow for seven days of recording. Their second baseline appointment included completion of a strategic processing task while EEG recorded electrical brain activity, followed by a behavioral measure of wayfinding performance. Following completion of all baseline measures, participants were randomized in a 1:1 ratio to the aerobic exercise with iPad group (experimental) or aerobic exercise only (control) group.

### 2.2. Exercise Protocol for All Participants

The 10-week intervention period was comprised of two supervised, moderate to vigorous aerobic exercise sessions (choice of recumbent stationary bike, elliptical, or treadmill) in which biweekly sessions took place at a local fitness facility. Weekly exercise sessions were scheduled with the aim of obtaining 20 total sessions. Exercise session dosage was titrated such that week one comprised of one 30-min and one 35-min session, week two included a 40-min and 45-min session, and by week three, participants exercised for 50 min per session and continued to do so for the subsequent seven weeks. In addition to scheduled sessions, participants were encouraged to partake in unobserved, supplemental aerobic exercise in order to meet the minimum 150 min per week recommendation according to ACSM and AHA guidelines [45]. Resting heart rate was recorded at baseline with the following formula [(220 − age) − (resting heart rate) × 0.50 and 0.75] and was used to calculate target heart rate zones that were implemented during exercise. Participants’ heart rate was checked every five minutes through use of an oximeter while trained research assistants encouraged participants to stay within this range. At the conclusion of each session, we assessed perceptions of affect, effort, and engagement.

### 2.3. Intervention Conditions

#### 2.3.1. iPad-Enhanced Aerobic Exercise Condition (Experimental Group)

**Figure 2:**
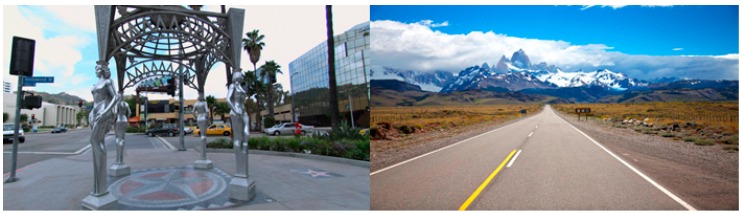
Urban and rural scenes from BitGym© application.

Participants in this condition engaged in a commercially available application (BitGym©) installed on an iPad positioned on their aerobic machine. BitGym© is an interactive application that delivers real 3D video of picturesque landscapes from locations around the world (see Figure 2). Each “tour” responds to the speed of the user through detection of bodily movement. Participants were asked to fully engage with each tour throughout the entire session. They were not allowed to listen to music or engage in any other activities (e.g., using smartphone, reading a magazine, doing a crossword puzzle) while exercising and they were discouraged from watching TV.

#### 2.3.2. Aerobic Exercise-Only Condition (Control Group)

Participants in this group completed each exercise session at the same prescribed dose (intensity, duration, frequency) as the experimental group. The control group had the same restrictions in terms of engaging in other activities during each session.

### 2.4. Primary Outcomes

#### 2.4.1. Wayfinding Self-Efficacy

Self-efficacy beliefs pertaining to confidence in one’s ability to navigate were assessed using established measures, which were unique from the screening questionnaire. Specifically, we used the Maze subscale of the Memory Self-Efficacy Questionnaire [46]. Example item: “If I had to find my way through a maze (on paper) on my first try, and the directions had 10 steps in them, I could find my way through part of the maze using all 10 steps in the directions.” Participants respond to five total items that are presented in a hierarchical order such that self-efficacy judgments are made at each level of task difficulty (i.e., using 10 steps, 8 steps, 6 steps, 4 steps, 2 steps) using a 0%–100% scale in 10% increments. The composite score was based on the average response of the five items.

#### 2.4.2. Wayfinding Task

The wayfinding task was adapted from previous research that utilized finding one’s way to a specific location in shopping malls [5,47,48] and targeted performance measures including time to complete the task, distance covered, and number of stops [49]. In this study, both objective and subjective measures were assessed. Specifically, participants completed a timed wayfinding task within a local six-story University building open to the community. Researchers predetermined a unique starting and ending location. A research assistant trailed closely behind each participant to record task completion time, as well as points of indecision or “hovers.” Hovers were defined as 3-s pauses made during the wayfinding task. Prior to beginning the assessment, participants were read a set of instructions, directing them to (a) use stairwells instead of elevators to access different floors, (b) avoiding interaction(s) with building patrons, (c) walk rather than run to complete the task, and (d) stop upon reaching the destination to allow research assistants to record Fitbit data. After instructions were read to participants, they were provided the floor and room number to find (e.g., room 338 on the third floor). Two pedometers were worn on participants’ hips to provide the most accurate estimate of number of steps and distance traveled (averages were used in analyses). At the end of the task, mood and perceived exertion levels were also assessed as an index of task difficulty. Mood was assessed using the Wong-Baker Face Scale [50], similar to that developed by Stern and colleagues and validated with neurological-impaired adults [51] where faces correspond to variations in mood ranging from happy (100%) to sad (0%). Perceived effort was assessed via a commonly used modification (0 to 10 rating) of Borg’s Ratings of Perceived Exertion (RPE) scale [52]. A maximum time limit was set at 15 min to complete the task. No participant exceeded this time limit. For the purposes of our primary analysis, we used total time to complete task as our performance index.

### 2.5. Secondary Outcomes

#### 2.5.1. Executive Function

Five measures were used to assess components of executive function. A dual-task paradigm assessed attentional load and working memory, the Stroop color-word test and Flanker Inhibitory Control test assessed inhibition, an experimental Strategic Processing task tested value-directed attention and inhibitory control, and Card Rotations and Hidden Patterns tests assessed visuospatial memory.

For the computerized dual-task paradigm, participants were instructed to respond to either a single-task (one stimuli) or a dual-task (two-stimuli) as quickly and accurately as possible. The single tasks involve the participant responding, using keys on the keyboard, to either two letters (A and B) or two numbers (2 and 3) in the center of the screen. If the letter A is presented, the participant presses the N key and if the letter B is presented, the participant presses the M key [53]. The second task consists of the numbers 2 (press the Z key) or 3 (press the X key). The dual task shows both a letter (A or B) and a number (2 or 3) on the screen simultaneously and the participant must respond to both stimuli. Participants were provided with one practice session, with the actual trial containing 48 trials. The outcome measures for the dual task are the accuracy and reaction time.

For the computerized Stroop task [54], participants were instructed to respond to the color (blue, red, or green) of each word presented on the screen as quickly and accurately as possible. There are four equally presented stimuli: congruent, incongruent-eligible, incongruent-ineligible, and neutral. The congruent stimuli consist of the meaning of the word matching the color of the word (RED in red color). The incongruent-eligible stimuli consist of the word being one of the potential color responses (RED in green color). The incongruent-ineligible stimuli consist of the word being a color, but not a potential response (BLACK in blue color). The neutral trial words have the same length and frequency of the response word, but not a color category (DOG in red). The outcome measures include an accuracy and reaction time score for the Stroop task.

The Flanker task consisted of a series of five arrows, in which participants were instructed to respond to the direction of the center arrow: whether it was congruent (>>>>>) or incongruent (>><>>). The participant responded to the center arrow facing left by pressing the X key and responded to the center arrow facing right by pressing the M key on the keyboard [55]. The participant was provided one practice session where feedback for correct response and reaction time were displayed for each trial. The actual session did not provide feedback. Accuracy and reaction time were recorded for this task.

#### 2.5.2. Strategic Processing Task

Participants completed a strategic processing task [56,57], which is a value-directed word list-learning task. The task consists of five-word lists each with a unique set of 40 words. In each list, half of the words (*n* = 20) were assigned to the high-value condition (worth 10 points) and half (*n* = 20) were assigned to the low-value condition (worth 1 point). High- and low-value words were differentiated by letter case, where words were either written in all uppercase letters (e.g., LAMB) or all lowercase letters (e.g., lamb). For each list, words were presented one at a time and EEG was simultaneously recorded. Participants were instructed to recall as many words as possible after each list with the goal of maximizing their score. The behavioral data in terms of number of high-value versus low-value words recalled were analyzed as metrics for value-directed strategic attention and inhibitory control.

#### 2.5.3. Spatial Memory

The Card Rotations test and Hidden Patterns test [58,59] were both used to assess visuospatial memory performance. Both tests are delivered via paper and pencil and are considered to be measures of spatial working memory such that participants are instructed to hold one particular “card” or “pattern” in mind while identifying correct rotations of that card depending on its orientation or pattern hidden within other patterns. Correct responses were added for each task, and then further added together to create a composite spatial memory performance score.

#### 2.5.4. Exercise Effort, Engagement, Attentional Focus and Affect

Perceived effort exerted during the exercise sessions was assessed with the modified RPE scale. Perceived engagement was assessed using an adapted version of the Game Engagement Questionnaire (GEQ) [60]. This questionnaire consisted of 14 items where participants responded to items reflecting states of absorption, flow, presence, and immersion using a 3-point Likert scale (0 = No, 1 = Sort of, 2 = Yes). Composite scores were calculated by summing participants’ responses to each subscale. Additionally, attentional focus (AF) was assessed via a single item on an 11-point Likert (0 = internal thoughts, 10 external thoughts) [61]. Perceptions related to acceptability and enjoyment were derived from the 20-item Positive and Negative Affect Scale (PANAS [62]). The PANAS questionnaire is divided into two subscales, one indicating positive or pleasant states, and one indicating negative or distressed states. Both subscales are calculated through summation of respective items on a 5-point Likert scale (1 = Very slightly or not at all, 2 = A little, 3 = Moderately, 4 = Quite a bit, 5 = Extremely) where high values represent greater positive or negative affective states. Higher composite scores on each subscale represent greater negative affect or positive affect. RPE, GEQ, AF, and PANAS were assessed immediately following each session with minimal delays (e.g., bathroom break). The first four sessions (weeks 1 through 2) and last four sessions attended were averaged to allow for comparisons between the beginning and end of the trial. For most participants, this was weeks 9 through 10; data from last sessions attended were used for dropouts.

#### 2.5.5. Physical Activity and Mobility

To assess rates of physical activity, participants were instructed to wear a Fitbit Ultra device, a reliable and valid device for activity monitoring [63] for seven consecutive days pre- and post-intervention. Participants were provided a Fitbit at their initial baseline testing appointment and were instructed to wear their Fitbit during all waking hours with the exception of bathing time and any activity involving water (e.g., swimming), as the devices were not waterproof. After seven consecutive days of wear, participants returned their Fitbit at either their convenience or second baseline testing appointment. The same procedure was completed at follow-up. Two participants withdrew from the intervention; therefore, multiple imputation procedures were performed for missing data. Due to various reasons, three participants did not wear their Fitbit for seven consecutive days at baseline or follow-up. In order to aggregate seven complete days’ worth of data for these participants, a window of 10 days was considered such that seven days’ worth of data could be derived.

A battery of functional fitness measures assessing mobility, agility, body strength, and balance was carried out at baseline and follow-up. Functional mobility was assessed using the timed “Up and Go” task (TUG) [64], where participants are instructed to rise from a seated position, walk three meters, turn around a cone, and walk back to the chair and sit down, as quickly as possible. Scores on the TUG task have good interrater reliability and have been associated with executive function [65]. Additionally, Novak and colleagues recommend using the stair climb task as a benchmark against which physical impairments in older adults can be explored. This task’s degree of difficulty may be suggestive of greater cognitive load associated with the task allowing performance to be used as a predictor of cognitive decline and MCI. Participants completed the stair climb task by ascending a stairwell as quickly as possible, making sure to step single-footedly on each step (i.e., double stepping was not allowed). Time to complete this task was recorded.

### 2.6. Statistical Analyses

Analyses were conducted using SPSS 25 (SPSS Inc., Chicago, IL, USA) [66]. Baseline characteristics were analyzed to assess differences between the exercise with iPad group (Experimental) and exercise only group (Control). We assessed groupwise differences in normally distributed variables using analysis of variance (ANOVA) testing. Composite scores were calculated for primary and secondary outcomes as well as any covariates included in analyses. An a priori power analysis with G*Power 3.1.9.2 (Heinrich-Heine-Universität Düsseldorf, Düsseldorf, Germany) was conducted to estimate sample size necessary to detect change for the three primary outcomes (corrected *p*-value for statistical significance for the three comparisons was *p* < 0.017). To detect a moderate effect (f = 0.20) with a minimum of 80% power, for a 2 (group) × 2 (time) repeated measures ANOVA within-between interaction, with an assumed correlation of ≥0.80 between repeated measures, it was estimated that we needed a sample size of 32 (16 per group). Therefore, our pilot study analyses were underpowered to detect change as a function of our experimental group. Difficulties with recruitment caused us to fall short of our projected enrollment. All secondary statistical analyses (ANOVAs, *t*-tests) reported herein were exploratory due to the preliminary nature of our trial and should be interpreted with caution as any group differences (or lack thereof) could be influenced by Type I/II error. Note that data were assumed to be missing at random and established multiple imputation procedures based on expectation-maximization algorithm were conducted for the small portion of data missing at baseline and follow-up. Specifically, five datasets of imputations were generated, averaged, and estimated values replaced missing cases in our dataset. No differences were revealed between data gathered from our imputation model and our existing dataset. Therefore, we proceeded with our primary data analysis.

## 3. Results

### 3.1. Feasibility and Acceptability

Overall, participants attended 98.70% of the twice-weekly, 50-min supervised sessions, and there was no difference between the intervention (*M* = 98.21 [*SD* = 4.64]) or control (*M* = 99.23 [*SD* = 1.88]) and participants maintained the prescribed moderate intensity with no adverse events reported. In addition, 76.79% were adherent to the full exercise prescription by engaging in an additional 50 min of unsupervised physical activity per week over the course of the 10-week program (meeting minimum public health guidelines for physical activity). There were no group differences in perceived effort (RPE), or engagement as measured by the single AF item at the beginning (overall sample *M* = 4.24 [*SD* = 1.99]) or end of the trial (*M* = 4.56 [*SD* = 2.98]), which implies that participants’ level of physical and mental effort was consistent across groups. AF scores reflected a somewhat “balanced” focus midway between internal and external thoughts. Interestingly, the “presence” (a subscale of the GEQ) was higher *(independent t-test*, *p* = 0.016) for the experimental group in the last session (*M* = 1.48, *SD* = 0.62 vs. *M* = 0.82, *SD* = 0.69) relative to the control group. Composite scores on the PANAS subscales were not statistically different between groups. Overall, there was a favorable positive-to-negative score ratio across the duration of the trial. Specifically, the sample averages for positive affect were 32.87 (*SD* = 7.88) at baseline and 33.43 (*SD* = 9.14) at follow-up, whereas averages for negative affect were 11.42 (S*D* = 1.56) and 10.50 (*SD* = 0.86), respectively. Together, these data suggest that the exercise program was well-received by our entire sample.

### 3.2. Primary Outcomes

No significant group differences were observed at baseline on any primary outcome measure. ANOVA for wayfinding (maze) self-efficacy (adjusting for baseline score) yielded a group effect, *F* (1, 24) = 4.42, that did not reach statistical significance upon correcting for multiple comparisons (*p* = 0.046, partial η^2^ = 0.156). However, after adjusting for whether or not participants received full dose (trial completion) and baseline physical activity (Fitbit steps) that accounts for age and gender in its algorithm, the group effect was magnified (*F* (1, 22) = 7.61, *p* = 0.011, partial η^2^ = 0.257). Indeed, participants in the experimental group (*M* = 85.00, *SD* = 16.19) had higher self-efficacy than participants in the control group (*M* = 74.46, *SD =* 16.58) at the 12-week follow-up (Table 2).

All participants completed the novel wayfinding task at baseline. There were no significant differences between group’s in wayfinding performance time (Table 2). One participant withdrew from the trial and one participant injured their leg and therefore did not complete the wayfinding task at follow-up. With the exception of one participant, all were able to find their destination at both timepoints in under 10 min. On average, at baseline, participants rated the task between “moderate” to “somewhat hard” and they were generally happy overall (*M* = 77.04%, *SD* = 14.36%; no group differences at either time point). Non-significant correlations between performance on the TUG task and time to complete the wayfinding task at each measurement occasion indicate mobility was not a potential barrier (*r’s* = 0.01, 0.29, *p* > 0.05). In addition, correlations between right and left hip-worn Fitbits indicated negligible discrepancies (*r’s =* 0.98, 1.00, *p* < 0.05) in the step counts obtained at baseline and follow-up. Together, these data suggest that the task was optimally challenging and performance measures collected were reliable.

### 3.3. Secondary Outcomes

No significant differences were observed on any secondary outcome measure at baseline (Table 3). Reaction time for the incongruent condition of the Stroop task was faster at follow-up, *F* (1, 23) = 5.75, *p* = 0.025, partial η^2^ = 0.200, for the experimental group. This effect did not change after adjusting for trial completion or baseline physical activity.

The results did not indicate significant differences between groups on either of the two other assessments of executive functioning or spatial memory. No significant group differences were observed on the strategic processing task at follow-up.

## 4. Discussion

This study investigated wayfinding on a novel, real-world behavioral task following a 10-week aerobic exercise intervention. As part of the intervention, one group of participants engaged in iPad-enhanced aerobic exercise while the other group engaged in aerobic exercise only. Although we did not observe group differences for wayfinding or spatial memory performance, univariate analyses revealed significant group differences in self-efficacy for wayfinding as well as group differences in the Stroop task at follow-up. Given that this was a pilot study, and despite the fact that most of our analyses were exploratory and underpowered, the results are promising and align with prior research supporting older adults’ self-efficacy for wayfinding to be positively associated with self-reported walking behavior. Indeed, improvements in inhibitory control tasks (Stroop) after 10 weeks of exercise is supportive of the previously established relationship between increased aerobic exercise and improved cognitive function among adults [67]. It is important to note that this intervention appears to be feasible for and accepted by this population. Specifically, overall attendance to the supervised aerobic sessions was high and the majority of participants were adherent to the full exercise prescription (reaching 150 min of moderate intensity aerobic exercise per week). All the participants seemed to exert equal levels of effort and engagement and exhibited higher levels of positive affect relative to negative affect throughout the duration of the trial, suggesting that the addition of a virtual reality iPad program does not serve as a burden while exercising.

A systematic review of interactive cognitive-motor training interventions revealed evidence supporting their use in reducing physical and cognitive risk factors associated with falls [68]. Interactive cognitive-motor training requires information processing, attentional control, and planning while executing motor responses. While some studies have highlighted the relationship between cognition and wayfinding performance [5,51], the role of physical activity within this relationship has only recently been explored. Given the physical nature of wayfinding and its cognitive demands, it seems likely that an interactive, cognitively stimulating physical activity intervention would enhance wayfinding efficacy and performance. Indeed, a recent review of physical activity programs enriched with cognitive challenges found that combined physical activity and cognitive activity programs showed significantly larger gains in cognition relative to physical activity alone [69]. Specifically, these results appeared to be more pronounced for studies that used simultaneous designs compared to sequential designs. These results are consistent with the results found herein. It is important to note that the aerobic exercise was completed in the field, at a local fitness facility, and televisions were mounted from the ceiling. Participants were explicitly told to focus on their exercise and were discouraged from watching the televisions during the supervised sessions. However, some distraction (cognitive-motor training load) was present for both groups and may have contaminated our effects.

Although we did not find differences between the two groups for wayfinding task performance, several factors may have influenced these results. First, the wayfinding task took place within a university building and subtle changes to the environment (visual or auditory distractions) may have negatively influenced performance. However, this seems unlikely as there were no significant group differences in AM or PM, weekday vs. weekend, or average group testing times at baseline (3:45 p.m. [*SD* = 3.19 h]) or follow-up (4:38 p.m. [*SD* = 2.63 h]). Indeed, the building was selected because it has relatively low pedestrian traffic by students and faculty. It is perhaps more likely that our attempt to simulate real-life [indoor] wayfinding in a testing environment did not sufficiently match the indoor training modality (engagement with a virtual reality app comprised of interactive outdoor scenes), negating any potential transfer effects. Moreover, our inclusionary criteria allowed for considerable sample heterogeneity in terms of the etiology and severity of their cognitive impairment. Although our screening was intended to minimize variability, it is quite possible that individuals with recent brain injury or persistent effects from brain trauma (e.g., car accident, chemotherapy) may have received approval from their physicians to participate in our trial. Someone with a traumatic brain injury could have responded with a different trajectory relative to those experiencing normal age-related decline or early-stage neurodegenerative disease. Despite the presence of outliers in our dataset, sensitivity analyses did not reveal any consistent or conflicting patterns contradicting those reported herein.

Future studies should aim to better match training stimuli to the performance response-set, whereas assessments of training transfer should include a combination of virtual wayfinding and indoor and outdoor wayfinding assessments (e.g., corn maze) to determine the extent of environmental influences on performance. Secondly, the relationship between cognitive-motor functions and strategic processing abilities is unknown and there may not have been sufficient measurement sensitivity and overlap between the targeted outcomes of the strategic processing task (recalling words of varied value) with the wayfinding intervention and performance task due to which no significant group effects were found. It is also possible that the dose of the intervention received was sufficient for eliciting change in certain cognitive areas (e.g., inhibition assessed via Stroop) but not for others (e.g., strategic processing and memory). To better understand the efficacy of the intervention for promoting wayfinding and cognitive function, other relevant memory tasks (“recall the order of these landmarks”), and “find your way” scenarios tasks should be considered.

## 5. Conclusions

The global older adult population is expected to exponentially increase over the next decade, and the prevalence of cognitive impairment is also projected to rise [70]. Therefore, interventions designed to prevent and slow the progression of cognitive impairment among aging adults are needed. Although “real life transfer” did not occur in this study, we did see changes in the perceptions of one’s abilities as well as changes in cognition (via Stroop task), suggesting that the use of technology-enhanced aerobic exercise training is a research area worthy of further investigation. Wayfinding is linked to one’s willingness to walk around and independently explore their surroundings, thus, it deserves greater attention to promote independence as we age. Wayfinding requires attention and spatial memory, and impairments resulting from chronic conditions (e.g., MCI or AD) can deplete one’s resources for successfully navigating one’s life-space. Our preliminary results suggest that virtual technology and digital wayfinding games may be promising tools for promoting and preserving confidence to engage in day-to-day wayfinding. Future research is needed to determine the most efficacious ways to improve wayfinding performance.

## Figures and Tables

**Table 1:** Demographic characteristics (*N* = 27).

Characteristic	Intervention (*n* = 14)	Control (*n* = 13)
Age (*M* (*SD*))	49.36 (5.08)	53.23 (4.71)
Sex (% female)	85.7	69.2
Race (%)		
White	64.3	69.2
Black or African American	21.4	15.4
Asian	7.1	7.7
American Indian	0.0	7.7
Decline Response	7.1	0.0
Ethnicity (%)		
Not Hispanic or Latino	78.6	92.3
Hispanic or Latino	21.4	7.7
Education (% college degree)	85.7	76.9
BMI (*M* (*SD*))	31.75 (8.46)	32.49 (16.70)
TICS (*M* (*SD*))	21.07 (2.53)	21.46 (1.13)
GDS (*M* (*SD*))	0.43 (0.85)	0.69 (1.03)

Note. BMI, Body Mass Index. TICS, Telephone Interview for Cognitive Status. GDS, Geriatric Depression Scale.

**Table 2:** Wayfinding performance and self-efficacy means (SD) between groups.

Wayfinding Performance Measure	Conditions			
	Experimental (*n* = 14)		Control (*n* = 13)	
	Baseline	Follow-up	Baseline	Follow-up
Total steps	840.36 (358.89)	1071.89 (507.00)	968.54 (469.50)	997.77 (719.22)
Distance covered (miles)	0.18 (0.08)	0.24 (0.11)	0.21 (0.09)	0.21 (0.15)
Total time (seconds)	245.58 (117.90)	315.67 (169.15)	298.69 (144.44)	300.12 (204.40)
Hovers	2.14 (2.98)	1.97 (1.87)	4.08 (4.17)	1.38 (1.89)
Mood	80.00% (11.09%)	64.17% (21.51%)	73.85% (17.10%)	69.23% (17.54%)
Exertion	3.21 (1.72)	3.70 (2.05)	3.23 (1.92)	3.77 (1.48)
Familiarity (Yes/No)	4/10		4/10	
Maze SE	58.29 (18.73)	85.00 * (16.19)	59.69 (21.12)	74.46 * (16.58)

Note. * *p* < 0.05; Maze SE refers to self-report self-efficacy measure.

**Table 3:** Cognitive outcome measures by group.

Cognitive Task	Experimental (*n* = 14)		Control (*n* = 13)	
	Baseline *M* (SD)	Follow-up *M* (SD)	Baseline *M* (SD)	Follow-up *M* (SD)
Stroop Task—RT	781.16 * (113.50)	735.78 * (105.75)	727. 20 (110.82)	753.96 (119.26)
Flanker Task—RT	627.62 (89.29)	616.0 (78.11)	615.92 (83.83)	593.67 (88.51)
Dual Task—RT of Second Selection	1585.77 (219.52)	1504.15 (256.07)	1563.50 (514.07)	1629.22 (234.17)
Hidden Figures (Summation)	161.93 (74.24)	200.10 (73.89)	155.08 (57.76)	202.62 (50.79)
Card Rotations (Summation)	100.93 (34.30)	114.36 (34.48)	97.31 (25.81)	112.15 (26.59)

Note. * *p* < 0.05. Reaction time (RT) was recorded in milliseconds. Summation scores were derived from the total number of items correct in 3 min.

## References

[1] Vandenberg A.E. (2016). Human Wayfinding: Integration of Mind and Body. Community Wayfinding: Pathways to Understanding.

[2] Spiers H.J., Maguire E.A. (2008). The dynamic nature of cognition during wayfinding. J. Environ. Psychol..

[3] Mullen S.P., Palac D.E., Bryant L.L. (2016). Maps to apps: Evaluating Wayfinding Technology. Community Wayfinding: Pathways to Understanding.

[4] Passini R. (1981). Wayfinding: A conceptual framework. Urban. Ecol..

[5] Chebat J.-C., Gélinas-Chebat C., Therrien K. (2005). Lost in a mall, the effects of gender, familiarity with the shopping mall and the shopping values on shoppers’ wayfinding processes. J. Bus. Res..

[6] Taillade M., Sauzéon H., Dejos M., Arvind Pala P., Larrue F., Wallet G., Gross C., N’Kaoua B. (2013). Executive and memory correlates of age-related differences in wayfinding performances using a virtual reality application. Aging Neuropsychol. Cogn..

[7] Taillade M., Sauzéon H., Pala P.A., Déjos M., Larrue F., Gross C., N’Kaoua B. (2013). Age-related wayfinding differences in real large-scale environments: Detrimental motor control effects during spatial learning are mediated by executive decline?. PLoS ONE.

[8] Poranen-Clark T., von Bonsdorff M.B., Rantakokko M., Portegijs E., Eronen J., Pynnönen K., Eriksson J.G., Viljanen A., Rantanen T. (2017). The temporal association between executive function and life-space mobility in old age. J. Gerontol. Ser. A.

[9] Wiener J.M., Kmecova H., de Condappa O. (2012). Route repetition and route retracing: Effects of cognitive aging. Front. Aging Neurosci..

[10] Petersen R.C., Caracciolo B., Brayne C., Gauthier S., Jelic V., Fratiglioni L. (2014). Mild cognitive impairment: A concept in evolution. J. Intern. Med..

[11] Keynejad R.C., Marková H., Šiffelová K., Kumar N., Vlček K., Laczó J., Migo E.M., Hort J., Kopelman M.D. (2018). Spatial navigation deficits in amnestic mild cognitive impairment with neuropsychiatric comorbidity. Aging Neuropsychol. Cogn..

[12] Peter J., Sandkamp R., Minkova L., Schumacher L.V., Kaller C.P., Abdulkadir A., Klöppel S. (2018). Real-world navigation in amnestic mild cognitive impairment: The relation to visuospatial memory and volume of hippocampal subregions. Neuropsychologia.

[13] Ruggiero G., Iavarone A., Iachini T. (2018). Allocentric to egocentric spatial switching: Impairment in aMCI and Alzheimer’s Disease patients?. Curr. Alzheimer Res..

[14] Satariano W.A., Guralnik J.M., Jackson R.J., Marottoli R.A., Phelan E.A., Prohaska T.R. (2012). Mobility and aging: New directions for public health action. Am. J. Public Health.

[15] Onen F., Henry-Feugeas M.C., Roy C., Baron G., Ravaud P. (2008). Mobility decline of unknown origin in mild cognitive impairment: An MRI-based clinical study of the pathogenesis. Brain Res..

[16] Pettersson A., Olsson E., Wahlund L.-O. (2005). Motor function in subjects with mild cognitive impairment and early Alzheimer’s disease. Dement. Geriatr. Cogn. Disord..

[17] Mullen S.P., McAuley E., Satariano W.A., Kealey M., Prohaska T.R. (2012). Physical activity and functional limitations in older adults: The influence of self-efficacy and functional performance. J. Gerontol. Ser. B Psychol. Sci. Soc. Sci..

[18] Peel C., Baker P.S., Roth D.L., Brown C.J., Bodner E.V., Allman R.M. (2005). Assessing mobility in older adults: The UAB Study of Aging Life-Space Assessment. Phys. Ther..

[19] Curcio C.-L., Alvarado B.E., Gomez F., Guerra R., Guralnik J., Zunzunegui M.V. (2013). Life-Space Assessment scale to assess mobility: Validation in Latin American older women and men. Aging Clin. Exp. Res..

[20] Portegijs E., Rantakokko M., Mikkola T.M., Viljanen A., Rantanen T. (2014). Association between physical performance and sense of autonomy in outdoor activities and life-space mobility in community-dwelling older people. J. Am. Geriatr. Soc..

[21] Portegijs E., Iwarsson S., Rantakokko M., Viljanen A., Rantanen T. (2014). Life-space mobility assessment in older people in Finland; measurement properties in winter and spring. BMC Res. Notes.

[22] Lo A.X., Brown C.J., Sawyer P., Kennedy R.E., Allman R.M. (2014). Life-space mobility declines associated with incident falls and fractures. J. Am. Geriatr. Soc..

[23] Lautenschlager N.T., Cox K.L., Flicker L., Foster J.K., van Bockxmeer F.M., Xiao J., Greenop K.R., Almeida O.P. (2008). Effect of physical activity on cognitive function in older adults at risk for Alzheimer disease: A randomized trial. J. Am. Med..

[24] Kramer A.F., Erickson K.I. (2007). Capitalizing on cortical plasticity: Influence of physical activity on cognition and brain function. Trends Cogn. Sci..

[25] Ströhle A., Schmidt D.K., Schultz F., Fricke N., Staden T., Hellweg R., Priller J., Rapp M.A., Rieckmann N. (2015). Drug and exercise treatment of Alzheimer disease and mild cognitive impairment: A systematic review and meta-analysis of effects on cognition in randomized controlled trials. Am. J. Geriatr. Psychiatry.

[26] Gates N., Singh M.A.F., Sachdev P.S., Valenzuela M. (2013). The effect of exercise training on cognitive function in older adults with mild cognitive impairment: A meta-analysis of randomized controlled trials. Am. J. Geriatr. Psychiatry.

[27] Hillman C.H., Erickson K.I., Kramer A.F. (2008). Be smart, exercise your heart: Exercise effects on brain and cognition. Nat. Rev. Neurosci..

[28] Baker L.D., Frank L.L., Foster-Schubert K., Green P.S., Wilkinson C.W., McTiernan A., Plymate S.R., Fishel M.A., Watson G.S., Cholerton B.A. (2010). Effects of aerobic exercise on mild cognitive impairment: A controlled trial. Arch. Neurol..

[29] McAuley E., Kramer A.F., Colcombe S.J. (2004). Cardiovascular fitness and neurocognitive function in older adults: A brief review. Brain Behav. Immun..

[30] Colcombe S., Kramer A.F. (2003). Fitness effects on the cognitive function of older adults: A meta-analytic study. Psychol. Sci..

[31] Boulos M.N.K., Yang S.P. (2013). Exergames for health and fitness: The roles of GPS and geosocial apps. Int. J. Health Geographics.

[32] Anderson-Hanley C., Arciero P.J., Brickman A.M., Nimon J.P., Okuma N., Westen S.C., Merz M.E., Pence B.D., Woods J.A., Kramer A.F. (2012). Exergaming and older adult cognition: A cluster randomized clinical trial. Am. J. Prev. Med..

[33] Read J.L., Shortell S.M. (2011). Interactive games to promote behavior change in prevention and treatment. J. Am. Med..

[34] Guderian B., Borreson L., Sletten L., Cable K., Stecker T., Probst M., Dalleck L. (2010). The cardiovascular and metabolic responses to Wii Fit video game playing in middle-aged and older adults. J. Sports Med. Phys. Fit..

[35] Colombo M., Marelli E., Vaccaro R., Valle E., Colombani S., Polesel E., Garolfi S., Fossi S., Guaita A. Virtual Reality for Persons with Dementia: An Exergaming Experience. Proceedings of the International Symposium on Automation and Robotics in Construction.

[36] van der Kuil M., van der Ham I., Visser-Meily J. Game Technology in Cognitive Rehabilitation of Spatial Navigation Impairment. Proceedings of the International Conference on Virtual Rehabilitation.

[37] Thompson Coon J., Boddy K., Stein K., Whear R., Barton J., Depledge M.H. (2011). Does participating in physical activity in outdoor natural environments have a greater effect on physical and mental wellbeing than physical activity indoors? A systematic review. Environ. Sci. Technol..

[38] Anderson-Hanley C., Maloney M., Barcelos N., Striegnitz K., Kramer A. (2017). Neuropsychological benefits of neuro-exergaming for older adults: A pilot study of an interactive physical and cognitive exercise system (iPACES). J. Aging Phys. Act..

[39] Wall K., Stark J., Schillaci A., Saulnier E., McLaren E., Striegnitz K., Cohen B., Arciero P., Kramer A., Anderson-Hanley C. (2018). The enhanced interactive physical and cognitive exercise system (iPACESTM v2. 0): Pilot clinical trial of an in-home iPad-based neuro-exergame for mild cognitive impairment (MCI). J. Clin. Med..

[40] Mrakic-Sposta S., Di Santo S.G., Franchini F., Arlati S., Zangiacomi A., Greci L., Moretti S., Jesuthasan N., Marzorati M., Rizzo G. (2018). Effects of combined physical and cognitive virtual reality-based training on cognitive impairment and oxidative stress in MCI patients: A pilot study. Front. Aging Neurosci..

[41] Gadler E., Grassi A., Riva G., Wiederhold B.K., Riva G. (2009). A rehabilitation protocol for empowering spatial orientation in MCI. A pilot study. Annual Review of Cybertherapy and Telemedicine 2009: Advanced Technologies in the Behavioral, Social, and Neurosciences.

[42] Knopman D.S., Roberts R.O., Geda Y.E., Pankratz V.S., Christianson T.J., Petersen R.C., Rocca W.A. (2010). Validation of the telephone interview for cognitive status-modified in subjects with normal cognition, mild cognitive impairment, or dementia. Neuroepidemiology.

[43] Welsh K.A., Breitner J.C., Magruder-Habib K.M. (1993). Detection of dementia in the elderly using telephone screening of cognitive status. Neuropsychiatry Neuropsychol. Behav. Neurol..

[44] Castanho T.J.C. (2017). Cognitive Functioning From Normal Aging to Mild Cognitive Impairment: Combining Rapid Cognitive Testing and Informant Reports to Improve Screening in Clinical and Research Contexts. Ph.D. Thesis.

[45] Haskell W.L., Lee I.-M., Pate R.R., Powell K.E., Blair S.N., Franklin B.A., Macera C.A., Heath G.W., Thompson P.D., Bauman A. (2007). Physical activity and public health: Updated recommendation for adults from the American College of Sports Medicine and the American Heart Association. Circulation.

[46] Berry J.M., West R.L., Dennehey D.M. (1989). Reliability and validity of the Memory Self-Efficacy Questionnaire. Dev. Psychol..

[47] Dogu U., Erkip F. (2000). Spatial factors affecting wayfinding and orientation: A case study in a shopping mall. Environ. Behav..

[48] Chebat J.-C., Gélinas-Chebat C., Therrien K. (2008). Gender-related wayfinding time of mall shoppers. J. Bus. Res..

[49] Hölscher C., Meilinger T., Vrachliotis G., Brösamle M., Knauff M. (2006). Up the down staircase: Wayfinding strategies in multi-level buildings. J. Environ. Psychol..

[50] Wong D., Hockenberry-Eaton M., Wilson D., Winkelstein M., Schwarts P., Schwarts P. (2001). Face scale. Wong. Face Facets P. Face.

[51] Stern R., Arruda J., Hooper C., Wolfner G., Morey C. (1997). Visual analogue mood scales to measure internal mood state in neurologically impaired patients: Description and initial validity evidence. Aphasiology.

[52] Burdon J.G.W., Juniper E.F., Killian K.J., Hargreave F.E., Campbell E.J.M. (1982). The perception of breathlessness in asthma. Am. Rev. Respir. Dis..

[53] Wong C.N., Chaddock-Heyman L., Voss M.W., Burzynska A.Z., Basak C., Erickson K.I., Prakash R.S., Szabo-Reed A.N., Phillips S.M., Wojcicki T. (2015). Brain activation during dual-task processing is associated with cardiorespiratory fitness and performance in older adults. Front. Aging Neurosci..

[54] Prakash R.S., Erickson K.I., Colcombe S.J., Kim J.S., Voss M.W., Kramer A.F. (2009). Age-related differences in the involvement of the prefrontal cortex in attentional control. Brain Cogn..

[55] Kramer A.F., Humphrey D.G., Larish J.F., Logan G.D. (1994). Aging and inhibition: Beyond a unitary view of inhibitory processing in attention. Psychol. Aging.

[56] Castel A.D., Humphreys K.L., Lee S.S., Galván A., Balota D.A., McCabe D.P. (2011). The development of memory efficiency and value-directed remembering across the life span: A cross-sectional study of memory and selectivity. Dev. Psychol..

[57] Nguyen L.T., Marini F., Zacharczuk L., Llano D.A., Mudar R.A. (2019). Theta and alpha band oscillations during value-directed strategic processing. Behav. Brain Res..

[58] Ekstrom R., French J. (1976). The Hidden Patterns Test. Educational Testing Service.

[59] Ekstrom R., French J., Harman H., Derman D. (1976). Card Rotations Test. Kit of Factor-Referenced Cognitive Tests.

[60] Brockmyer J.H., Fox C.M., Curtiss K.A., McBroom E., Burkhart K.M., Pidruzny J.N. (2009). The development of the Game Engagement Questionnaire: A measure of engagement in video game-playing. J. Exp. Soc. Psychol..

[61] Tenenbaum G., Connolly C.T. (2008). Attention allocation under varied workload and effort perception in rowers. Psychol. Sport Exerc..

[62] Watson D., Clark L.A., Tellegen A. (1988). Development and validation of brief measures of positive and negative affect: The PANAS scales. J. Personal. Soc. Psychol..

[63] Adam Noah J., Spierer D.K., Gu J., Bronner S. (2013). Comparison of steps and energy expenditure assessment in adults of Fitbit Tracker and Ultra to the Actical and indirect calorimetry. J. Med. Eng. Technol..

[64] Podsiadlo D., Richardson S. (1991). The timed “Up & Go”: A test of basic functional mobility for frail elderly persons. J. Am. Geriatr. Soc..

[65] McGough E.L., Kelly V.E., Logsdon R.G., McCurry S.M., Cochrane B.B., Engel J.M., Teri L. (2011). Associations between physical performance and executive function in older adults with mild cognitive impairment: Gait speed and the timed “up & go” test. Phys. Ther..

[66] IBM Corp (2017). IBM SPSS Statistics for Windows.

[67] Kramer A., Erickson K.I., Colcombe S.J. (2006). Exercise, cognition and the aging brain. J. Appl. Physiol..

[68] Schoene D., Valenzuela T., Lord S.R., de Bruin E.D. (2014). The effect of interactive cognitive-motor training in reducing fall risk in older people: A systematic review. BMC Geriatr..

[69] Gheysen F., Poppe L., DeSmet A., Swinnen S., Cardon G., De Bourdeaudhuij I., Chastin S., Fias W. (2018). Physical activity to improve cognition in older adults: Can physical activity programs enriched with cognitive challenges enhance the effects? A systematic review and meta-analysis. Int. J. Behav. Nutr. Phys. Act..

[70] Matthews F.E., Stephan B.C., Robinson L., Jagger C., Barnes L.E., Arthur A., Brayne C., Collaboration A.S.C., Comas-Herrera A., Wittenberg R. (2016). A two decade dementia incidence comparison from the Cognitive Function and Ageing Studies I and II. Nat. Commun..

